# Loss of Wave1 gene defines a subtype of lethal prostate cancer

**DOI:** 10.18632/oncotarget.3564

**Published:** 2015-03-31

**Authors:** Adam G. Sowalsky, Rebecca Sager, Rachel J. Schaefer, Gennady Bratslavsky, Pier Paolo Pandolfi, Steven P. Balk, Leszek Kotula

**Affiliations:** ^1^ Department of Medicine, Beth Israel Deaconess Medical Center, Harvard Medical School, Boston, MA 02215, USA; ^2^ Department of Biochemistry and Molecular Biology, SUNY Upstate Medical University, Syracuse, NY 13210, USA; ^3^ Department of Urology, SUNY Upstate Medical University, Syracuse, NY 13210, USA; ^4^ Department of Pathology, Beth Israel Deaconess Medical Center, Harvard Medical School, Boston, MA 02215, USA; ^5^ Cancer Research Institute, Beth Israel Deaconess Cancer Center, Harvard Medical School, Boston, MA 02215, USA

**Keywords:** WAVE, prostate cancer, genomics, androgen receptor, castration resistance

## Abstract

Genetic alterations involving *TMPRSS2*-*ERG* alterations and deletion of key tumor suppressor genes are associated with development and progression of prostate cancer (PCa). However, less defined are early events that may contribute to the development of high-risk metastatic prostate cancer. Bioinformatic analysis of existing tumor genomic data from PCa patients revealed that WAVE complex gene alterations are associated with a greater likelihood of prostate cancer recurrence. Further analysis of primary *vs*. castration resistant prostate cancer indicate that disruption of WAVE complex gene expression, and particularly *WAVE1* gene (*WASF1*) loss, is also associated with castration resistance, where *WASF1* is frequently co-deleted with PTEN and resists androgen deprivation therapy (ADT). Hence, we propose that *WASF1* status defines a subtype of ADT-resistant patients. Better understanding of the effects of WAVE pathway disruption will lead to development of better diagnostic and treatment modalities.

## INTRODUCTION

As the most-common noncutaneous cancer in men worldwide [1], mechanisms contributing to development of prostate cancer at all stages of disease remain of high interest for both diagnosis and treatment of clinically-relevant disease. Consistent with the multi-hit hypothesis, several genes that control critical growth, survival, and/or apoptotic pathways [2] must be altered to lead to fully penetrant prostate cancer [3–5]. A wide body of literature has identified many complex genetic alterations involved in neoplastic transformation, including *TMPRSS2*-*ETS* (*ERG*) chromosomal translocations and deletion of tumor suppressor genes (*PTEN*, *TP53*, *RB1*, *NKX3–1*, and *CDKN1B*) [3–10]. Of these, selection for the deletion of *PTEN* (phosphatase and tensin homolog) occurs in approximately 30% of prostate cancers, as lowered levels of PTEN increase the availability of phosphatidyl-inositol 3, 4, 5 triphosphate (PIP3) for driving PI 3-kinase (PI3K)-dependent signaling, such as cellular growth pathways downstream of AKT (protein kinase B) [7, 11]. In addition, we previously implicated *ABI1* (*SSH3BP1*) as a putative tumor suppressor in prostate cancer [12] and have demonstrated that disruption of *Abi1* in the mouse prostate leads to prostatic intraepithelial neoplasia (PIN), but not to invasive prostate cancer [13]. We have therefore sought to find additional genes that cooperate with *ABI1* in prostate tumor progression.

In cells Abi1 is incorporated in the WAVE complex. In mammalian cells, several distinct WAVE complexes can form, depending on the isoform of proteins that is expressed [14]. Each WAVE complex assembles from ubiquitously expressed variants of 5 polypeptides: WAVE, Abelson interactor (Abi or SSH3BP1), Rac1-associated protein (Sra-1), Nck-associated protein (Nap), and Brk1 (HSPC300) [15–17]. Except for Brk1 [18], for which only one isoform exists, each of these proteins is a member of a protein family consisting of 2–3 genes in mammals including alternatively spliced isoforms of 10 genes [14, 19]: (i) *WASF1*, WAVE2 (*WASF2*), and WAVE3 (*WASF3*), (ii) *ABI1*, *ABI2*, and *ABI3*, (iii) Sra-1 and PIR121 (*CYFIP1* and *CYFIP2*), and (iv) Nap1 (*NCKAP1*) and Hem1 (*NCKAP1L1*). Functionally, WAVE complexes are major actin cytoskeleton regulatory factors that promote actin polymerization through an Arp2/3 dependent mechanism [20]. Other functions of WAVE complex or its components involve binding to variety of membrane receptors [21], intracellular signaling [22, 23], and transcription [22, 24]. WAVE complexes are involved in cell motility and migration, cellular adhesion, cell-to-cell communication, cell division, and immunological responses [17, 25–31]. Thus, WAVE complexes are involved numerous cellular functions requiring actin cytoskeleton reorganization and dynamics, processes with roles in tumor progression and metastasis [32].

Consistent with this, dysregulation of WAVE complex or its components is associated with human cancer [33–35] including prostate cancer [12, 36–39], as the WAVE pathway cross talks with the PI3K pathway. It has been shown previously that PIP3 can bind directly to WAVE protein [40], and we and others have demonstrated that the p85 regulatory subunit of PI3K interacts with Abi1 [23, 41]. Importantly, we have also shown that genetic inactivation of *Abi1* led to down-regulation of *WASF1* (and *WASF2* [42]) which in turn resulted in increased phosphorylation of Akt in murine prostate tissue [13]. To this effect, altered levels of WAVE complex genes would result in disruption of the WAVE complex's ability to regulate p85, and thus would phenocopy deletion of *PTEN* in mediating cancer progression. Therefore we hypothesize that prostate cancers select for cells with lower levels of WAVE complex proteins.

In patients with metastatic PCa, standard treatments involve either surgical or medical castration, which effectively prevents testicular testosterone from driving androgen receptor (AR) activation in the tumor cell [43]. The fact that most patients invariably relapse has led to profound interest in identifying mechanisms contributing to the development of castration-resistant prostate cancer (CRPC) and commercial production of inhibitors of adrenal sources of androgens and AR pathway inhibitors such as galeterone, abiraterone and enzalutamide [44–46]. However, the fact that *PTEN* is deleted or down-regulated in these cancers has intensified investigation of how PI3K contributes to androgen independence via a PTEN-dependent mechanism. Not surprisingly, simultaneous inhibition of both AR and PI3K effectively blocked growth of human PCa xenografts [47–49]. These data further reinforce the need to identify causative mechanisms of CRPC development, especially in the context of *PTEN* deletion.

Therefore, to assess whether prostate cancer progression to castration resistance is mediated by changes to WAVE complex, we performed bioinformatic meta-analyses on several published datasets, including mutation, copy number, and gene expression data accumulated as part of The Cancer Genome Atlas (TCGA), as well as other publicly-available datasets deposited in GEO from experiments performed at Beth Israel Deaconess Medical Center (BIDMC), Memorial Sloan Kettering Medical Center (MSKCC) and the University of Michigan (UMICH) [7, 10, 50]. By comparing results across independent cohorts, we observed shifts in the frequency of WAVE complex gene alterations, which suggest that WAVE complex disruption may be a putative driver of prostate tumorigenesis. Importantly, we observed that deletion of *WASF1*, the gene that codes for WAVE1, occurs more frequently with *PTEN* deletion in metastatic lethal *vs*. primary disease, suggesting that *WASF1* loss represents an aggressive of subtype of prostate cancer. It is thus possible that patients who harbor tumors with *WASF1* deletion may benefit from either earlier or more aggressive intervention.

## RESULTS AND DISCUSSION

**Figure 1:**
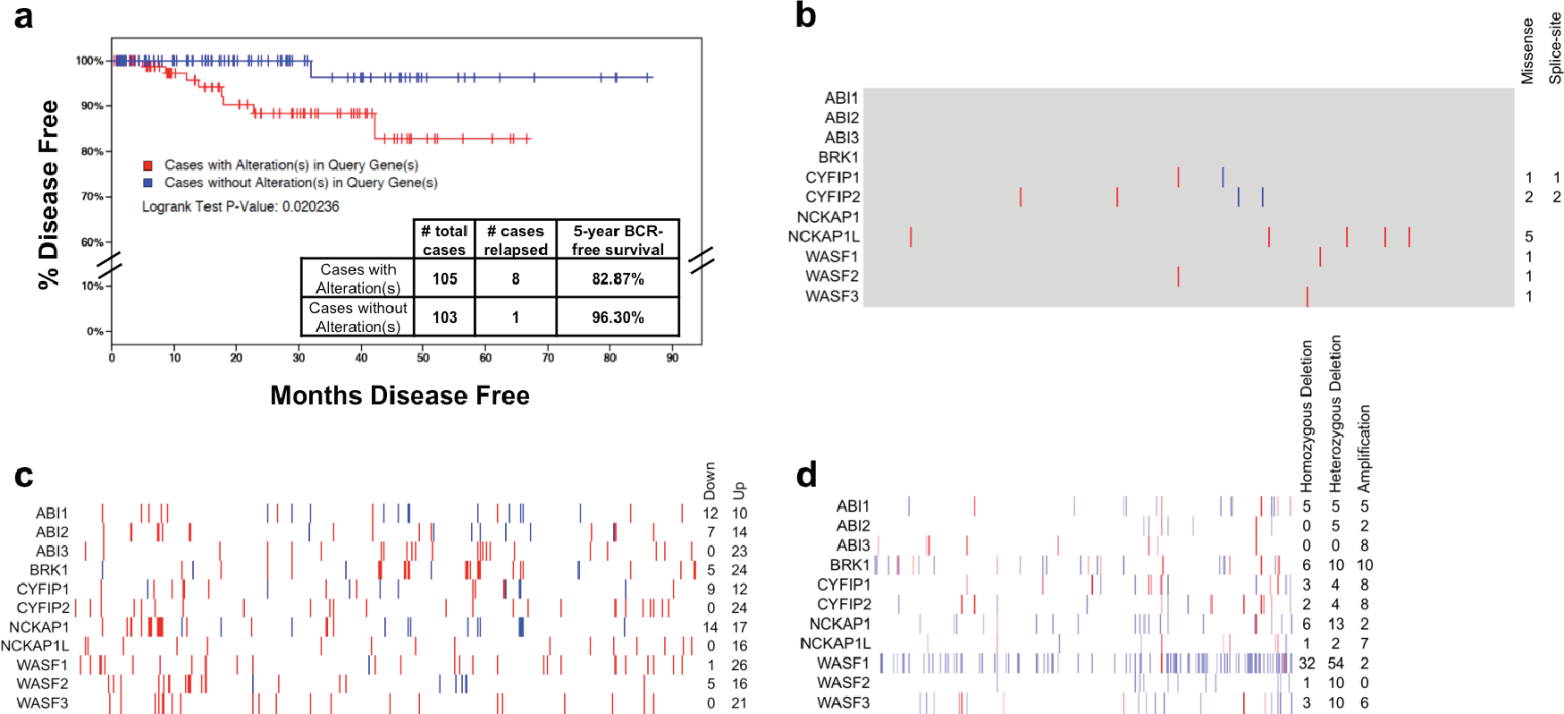
Spectrum of alterations of WAVE complex in primary prostate cancer Data on prostate adenocarcinoma analyses performed as part of The Cancer Genome Atlas (TCGA) as of the time of writing was downloaded from cBioPortal [64], the NCBI TCGA data portal (https://tcga-data.nci.nih.gov) and CGHub [65]. **a.** Kaplan-Meier survival distribution showing the time until biochemical recurrence for patients with alterations in WAVE complex genes (*ABI1*, *ABI2*, *ABI3*, *BRK1*, *CYFIP1*, *CYFIP2*, *NCKAP1*, *NCKAP1L*, *WASF1*, *WASF2*, and *WASF3*). Log rank *P* value: 0.020 suggesting a significant difference between groups. **b.** Distribution of nonsynonymous somatic mutations to WAVE complex genes from 493 cases of PCa whole exome sequencing data generated by the TCGA. Blue: splice-site mutation; red: missense mutation; gray: unchanged. **c.** Gene expression changes in tumor samples relative to the mean of the sample set's 75^th^ percentile-normalized RSEM values (*z*-score/standard deviations, s.d.) from 493 cases of TCGA PCa RNA-seq data [66]. Normalized RSEM values are given in Supplementary Table 1, and z-score values are given in Supplementary Table 2. Blue: ≥ 2 s.d. down-regulated; Red: ≥ 2 s.d. up-regulated. Note that due to variability in the purity of the tumor sample (Supplementary Table 3) as determined by ESTIMATE [67], some up- or down-regulation of WAVE complex genes may be due to stromal contamination. **d.** Somatic copy number analysis of WAVE complex genes from 319 cases of TCGA PCa Affymetrix 6.0 SNP arrays, where 1 (white) = two copies. Red: > 1.2, inferring amplification; Blue: < 0.8, inferring deletion. Homozygous deletion: copy number less than 0.6. Hemizygous deletion: copy number 0.6–0.8. Cases with tumor cell purity less than 90% are excluded from totals, but values for all cases are given in Supplementary Table 4.

We had previously assessed the status of *ABI1* in a small cohort of primary prostate tissue [51], so we expanded our interrogation of WAVE complex alterations to The Cancer Genome Atlas (TCGA) and molecular analyses performed on these samples. We hypothesized that alterations to the WAVE complex may contribute to increased tumorigenicity and therefore poorer long-term outcome. When we interrogated the dataset for cases with mutations, copy number alterations, or abnormal expression, we found that alterations to WAVE complex genes were significantly associated with a 13.43% increase in the rate of biochemical recurrence (BCR) within 5 years (Figure 1a). Interrogating other datasets for which biochemical recurrence rates were available, we observed a similar trend in the MKSCC dataset, where alterations to WAVE complex genes were associated with a 11.14% increase in the five-year BCR rate (Supplementary Figure 1). Strikingly, the most common alterations were not mutations (Figure 1b) nor recurrent up- or down-regulation (Figure 1c), but rather copy number variation to WAVE complex genes, preeminently WAVE1 (*WASF1*) (Figure 1d), which was deleted hemizygously in 54 cases (16.9%) and homozygously in 32 cases (10%).

These observations are consistent with several recent reports that describe many recurrent translocations and deletions in prostate cancer and surprisingly few recurrent mutations. Indeed, mutations to *SPOP, MED12* and *FOXA1* comprise less than 10% (each) recurrence in primary PCa exomes sequenced as part of TCGA and previous studies [52], but large chromosomal events occur far more frequently, with translocations (or interstitial deletions) involving *TMPRSS2* and *ETS*-family transcription factors (such as *ERG*) in approximately 50% of cases, or tumor suppressor deletions (*PTEN, NKX3–1*) in approximately 30–50% of cases [7]. Our observation of *WASF1* deletion was not as frequent (approximately 25%). Although these deletions were identified, these same cases did not indicate lower levels of *WASF1* mRNA. While we cannot rule out the possibility that cells can compensate by up-regulating the remaining copy, recent evidence suggests that the WAVE complex is regulated mostly at the protein level [42, 53].

**Figure 2:**
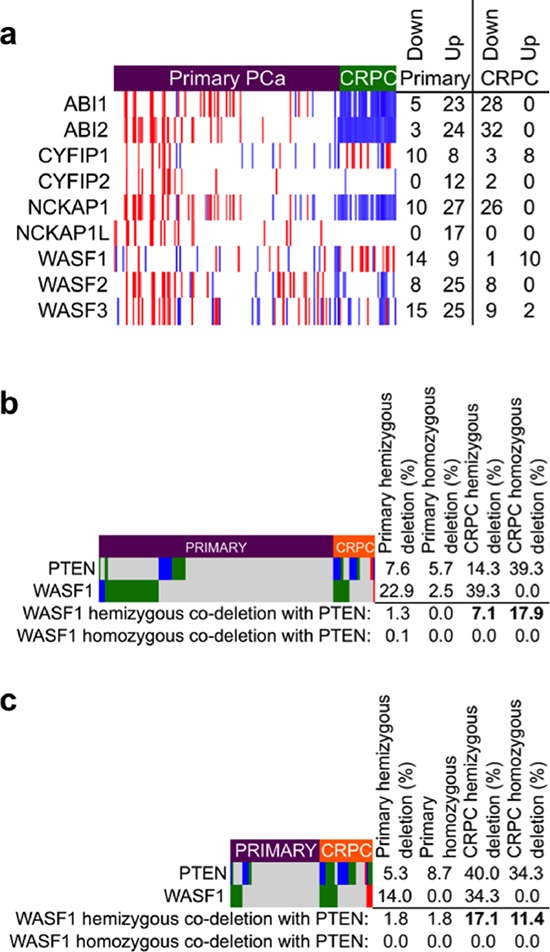
WAVE complex disruption in castration-resistant prostate cancer **a.** Heatmap of gene expression changes in WAVE complex genes relative to the mean of the sample. Microarray data for primary PCa (Affymetrix Human Exon 1.0 array, accession ID GSE21034) and CRPC (Affymetrix U133A array, accession ID GSE32269) were downloaded from the Gene Expression Omnibus (GEO) and normalized to the same scale using SCAN for Bioconductor [68]. Gene expression values in each row are displayed according to their *z*-score (number of standard deviations (s.d.) greater or lower than the mean for the sample set). Note that probes to *BRK1* and *ABI3* were not present on the Affymetrix U133A microarray and thus are excluded from this analysis. Red: high expression (greater than 1 s.d. up-regulated); blue: low expression (greater than 1 s.d. downregulated). Normalized intensity values are given in Supplementary Table 5 and z-scores are values are given in Supplementary Table 6. **b.** Somatic copy number depiction of *PTEN* and *WASF1* of 157 primary PCa and 28 CRPC from the MKSCC dataset [7] (Agilent 244A aCGH array, accession ID GSE21032). *P* < 0.0001 by Fisher's exact test for the frequency of co-occurrence of hemizygous *WASF1* deletion with combined frequencies of hemizygous and homozygous deletion of *PTEN* in CRPC *vs*. primary PCa. Numeric copy number calls are given in Supplementary Table 7. **c.** Somatic copy number depiction of *PTEN* and *WASF1* of 59 primary PCa and 35 lethal CRPC from the UMICH dataset [10] (Agilent 44K aCGH array, accession ID GSE35988). *P* = 0.0006 by Fisher's exact test for the frequency of co-occurrence of hemizygous *WASF1* deletion with combined frequencies of hemizygous and homozygous deletion of *PTEN* in CRPC *vs*. primary PCa. Numeric copy number calls are given in Supplementary Table 8. For (b) and (c) data were downloaded from GEO and loci copy number were assessed with Nexus Copy Number software (Biodiscovery, Hawthorne, CA); dark blue: homozygous deletion; green: hemizygous deletion; red: amplification; gray: unchanged.

Nonetheless, the increased frequency of biochemical recurrence of tumors with WAVE complex gene alterations and our observation of recurrent deletion of *WASF1* in primary prostate cancer therefore led us to ask whether WAVE complex was similarly disrupted in advanced PCa, in a castration-resistant (CRPC) setting. Examination of gene expression microarrays from 131 primary PCa (MSKCC dataset [7]) and 33 CRPC (BIDMC dataset [50]) revealed marked down-regulation of *ABI1*, *ABI2*, and *NCKAP1* in the CRPC samples (Figure 2a). *WASF1* expression displayed modest variation (within and between sets), which is consistent with the observation from TCGA (see Figure 1c) that *WASF1* down-regulation by itself is not a mechanism of progression in PCa, although down-regulation of *ABI1*, *ABI2*, or *NCKAP1* may lead to destabilization of WAVE complex and thus lower levels of WAVE1 protein.

Importantly, the WAVE complex acts as a mediator PI-3 kinase-directed cell motility, and thus may also contribute to overall tumorigenenicity when dysregulated [54, 55]. Given its function downstream of PI3K, we therefore sought to explore *WASF1* copy number status in the context of *PTEN* deletion, a frequent genomic lesion found in primary and advanced PCa [56–58]. To make comparisons, we selected two published datasets (from MSKCC [7] and UMICH [10]) in which copy number analysis was performed simultaneously in both primary PCa and CRPC. As anticipated, *PTEN* and *WASF1* were frequently deleted (hemizygously and homozygously) in primary PCa from both datasets (Figures 2b and 2c). To our surprise however, *PTEN* and *WASF1* deletion were mutually exclusive in primary PCa, but hemizygous *WASF1* deletion co-occurred with both hemizygous and homozygous *PTEN* deletion in CRPC (*P* < 0.0001 for the MSKCC set, *P* = 0.0006, for the UMICH set). As *PTEN* and *WASF1* are on different chromosomes (10q23 and 6q21) these events are not physically linked, and because in CPRC nearly all *WASF1* deletions were in cases that also harbored at least one copy of *PTEN* (but not *vice versa*), these data suggest that *WASF1* deletion is an earlier event preceding tumor cells’ acquisition of *PTEN* deletions that likely occur later as tumors progress.

**Figure 3:**
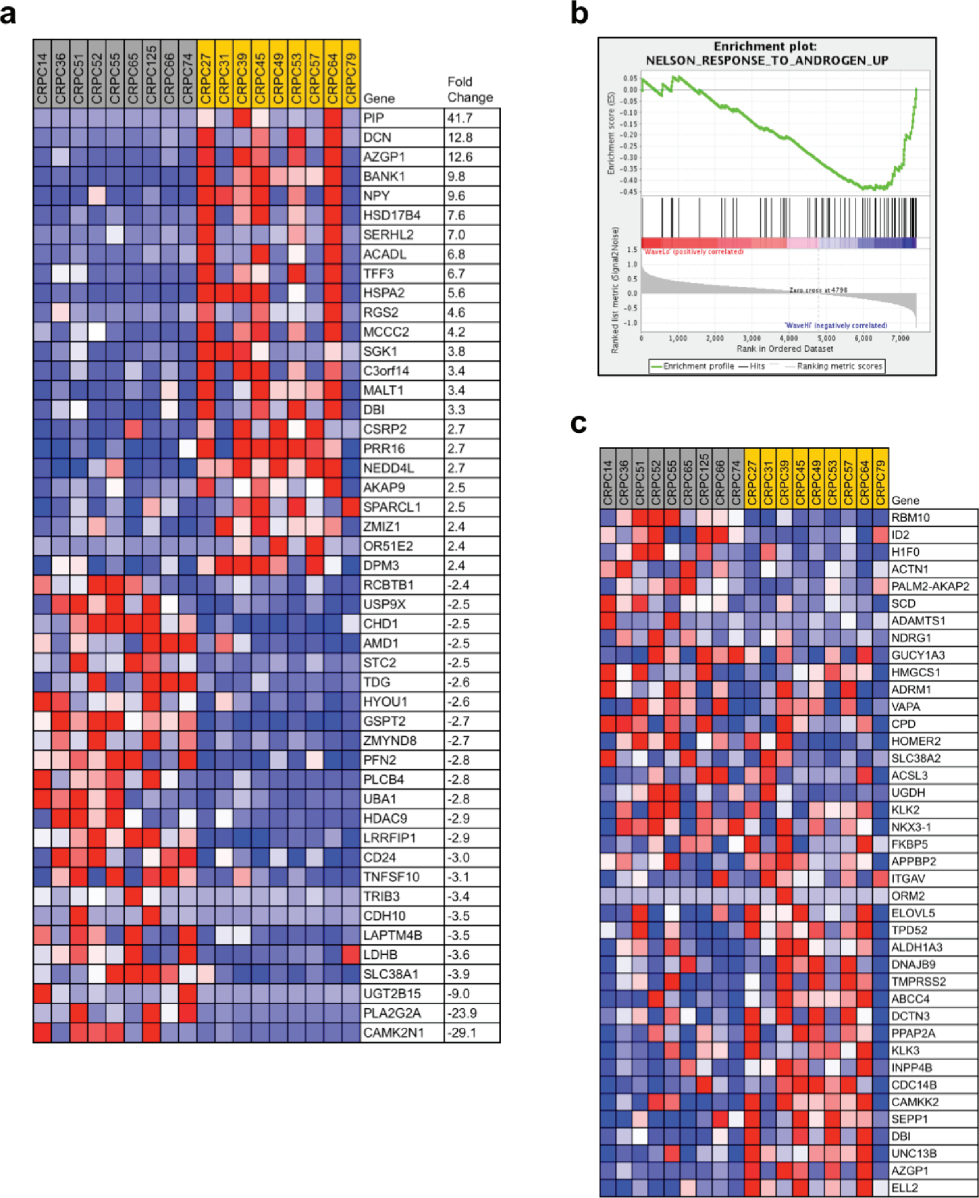
*WASF1* deletion may identify an aggressive subtype of prostate cancer **a.** Of the 36 CRPC samples in the BIDMC dataset (Affymetrix U133A array, accession ID GSE32269), the 9 samples with the highest quartile of *WASF1* expression (gray, mean = 2.67) were compared against the 9 samples with the lowest quartile of *WASF1* expression (yellow, mean = 1.41), and the genes contributing most to the distinguishing of these groups were identified with the ComparativeMarkerSelection [69] module in GenePattern [70] (fold-change ≥ ± 2.4 ×, *P* < 0.05 by Student's *t*-test). Red: high expression; blue: low expression. Expression intensity values are given in Supplementary Table 9. **b.** Enrichment plot of *WASF1*-high *vs. WASF1*-low gene expression and correlation with Androgen Receptor (AR) activity. Normalized enrichment score: –1.50; *P* = 0.038. **c.** Heatmap of gene expression associated with AR activity positively correlated with the *WASF1*-low group. Red: high expression; blue: low expression. Expression intensity values are given in Supplementary Table 10.

Importantly, as *PTEN* and *WASF1* were rarely deleted simultaneously in the same case in primary PCa (see Figures 2b and 2c), selection for dual loss may be associated with processes that mediate disease progression. Specifically, the frequency of *WASF1* deletion in lethal prostate cancer and its co-deletion with *PTEN* raised the possibility that advanced PCa cells select for *PTEN* deletion in cases with *WASF1* already deleted in order to drive PI 3-kinase signaling in castration-resistant cases with stronger AR reactivation. Furthermore, because we did not observe frequent homozygous deletion of *WASF1*, tumors with lower WAVE1 levels (rather than total loss of WAVE1) may similarly confer a selective advantage at later stages of disease. Indeed, *WASF1* expression within the BIDMC dataset of CRPC was varied to permit stratification of samples by the top quartile of *WASF1* expression (*WASF1*-high) *vs*. the lower quartile (*WASF1*-low) to predict the potential effects of *WASF1* genomic deletion (Figure 3a). Consistent with our hypothesis that low *WASF1* expression represents an aggressive phenotype, we observed significant down-regulation of the putative tumor suppressors *CHD1* and *CDH10* (cadherin-10) [59, 60]. Moreover, amongst the genes most up-regulated in the *WASF1*-low group are *HSD17B4*, which codes for an androgen inactivating enzyme [61], and *PIP* (prolactin-induced protein), whose expression was recently reported as a readout of AR (Androgen Receptor) activity [62]. Therefore, to test whether AR activity is indeed increased in the *WASF1*-low cases, we performed Geneset Enrichment Analysis (high *vs*. low) using the expression values for all genes. In the *WASF1*-low phenotype, we observed negative enrichment for up-regulated genes derived from a dataset in which LNCaP cells were treated with methyltrienolone/R1881 (Figure 3b) [63]. Indeed, these genes include the AR targets *KLK3* (PSA)*, TMPRSS2*, and *FKBP5*, which are up-regulated in the *WASF1*-low group (Figure 3c), suggesting that CRPC with lower levels of *WASF1* have increased tumorigenicity.

While metastatic CRPC circumvents androgen deprivation by intratumoral testosterone synthesis [50], increased expression of AR target genes suggests higher stability of ligand-bound AR and thus higher levels of androgen synthesis. Because the reactivation of AR activity and down-regulation of additional tumor suppressors in these cases (see Figure 3a) represents an aggressive prostate cancer phenotype, deletion of *WASF1* and lower levels of *ABI1* and *ABI2* likely cooperate with other perturbations in these CRPC for overcoming androgen deprivation, providing a selective advantage for those tumors harboring these genetic changes. Thus, the normal function of the WAVE complex may be to serve as a tumor suppressor. Therefore, *WASF1* deletion co-occurring with *PTEN* in advanced prostate cancers may result in even stronger PI3K signaling, having removed inhibitory forces from p85 (*WASF1* deletion) and increasing levels of PIP3 (*PTEN* deletion). Further investigation is needed to determine the biological consequences of *WASF1* deletion, the interactions between WAVE pathway and the AR signaling axis, and the role of WAVE pathway in establishing tumors with long-term aggressive potential.

## SUPPLEMENTARY FIGURE AND TABLES






















