# Supplementary material for: Loss of Wave1 gene defines a subtype of lethal prostate cancer
Source: Oncotarget. 2015 Mar 31;6(14):12383–91. doi: 10.18632/oncotarget.3564 (PMC4494945; doi:10.18632/oncotarget.3564)
Supplement: Supplementary file 1 [file oncotarget-06-12383-s001.pdf]

SUPPLEMENTARY FIGURE AND TABLES

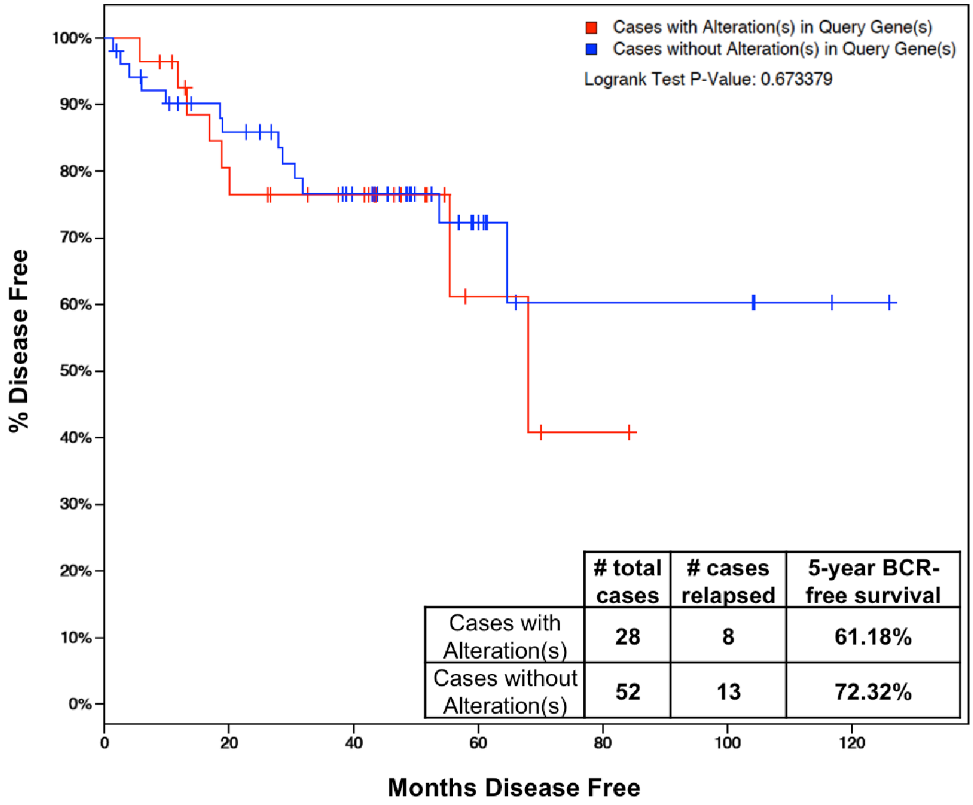

Supplementary Figure 1: Alterations in WAVE complex genes in tumors increase the rate of biochemical recurrence within five years. Kaplan-Meier survival distribution showing the time until biochemical recurrence for patients with alterations in WAVE complex genes (*ABI1*, *ABI2*, *ABI3*, *BRK1*, *CYFIP1*, *CYFIP2*, *NCKAP1*, *NCKAP1L*, *WASF1*, *WASF2*, and *WASF3*) from the MSKCC data set.

Supplementary Table 1: Normalized RSEM values for WAVE complex genes from RNA-seq in prostate TCGA cases. RSEM values were self-normalized against the 75th percentile of all RSEM values within each case.

Supplementary Table 2: *z*-score values for gene expression variance of WAVE complex genes from RNA-seq in prostate TCGA cases. *z*-scores were determined by subtracting each normalized RSEM value from the mean for the sample set, and dividing that difference by the standard deviation for each gene (across the sample).

Supplementary Table 3: Tumor cell purity determined by gene expression of prostate TCGA cases. ESTIMATE was used to apply 141-gene signatures for stromal cell contamination and immune cell contamination to infer a tumor cell purity (percentage).

Supplementary Table 4: Copy number values for 487 TCGA prostate cases. A value of 1 indicates normal heterozygosity (two copies).

Supplementary Table 5: Gene expression values for the MSKCC (primary PCa) and BIDMC (CRPC) datasets. SCAN was used to normalize both microarray platforms to the same scale.

Supplementary Table 6: *z*-score values for gene expression variance across the MSKCC (primary PCa) and BIDMC (CRPC) datasets. *z*-scores were determined by subtracting each gene value from the mean for the sample set, and dividing that difference by the standard deviation for each gene (across the sample).

Supplementary Table 7: Copy number calls for the MSKCC dataset. –2 indicates homozygous deletion. –1 indicates hemizygous deletion. 0 indicates no copy number change. 1 indicates copy number gain. 2 indicates high amplification.

Supplementary Table 8: Copy number calls for the UMICH dataset. –2 indicates homozygous deletion. –1 indicates hemizygous deletion. 0 indicates no copy number change. 1 indicates copy number gain. 2 indicates high amplification.

Supplementary Table 9: Gene expression values for the genes determined by GenePattern to contribute most to the *WASF1*-high vs. *WASF1*-low phenotypes. Values represent SCAN-normalized intensities.

Supplementary Table 10: Gene expression values for the AR activity geneset. Values represent SCAN-normalized intensities.
